# Serum Metabolomics Benefits Discrimination Kidney Disease Development in Type 2 Diabetes Patients

**DOI:** 10.3389/fmed.2022.819311

**Published:** 2022-05-09

**Authors:** Xiaofeng Peng, Xiaoyi Wang, Xue Shao, Yucheng Wang, Shi Feng, Cuili Wang, Cunqi Ye, Jianghua Chen, Hong Jiang

**Affiliations:** ^1^Kidney Disease Center, The First Affiliated Hospital, College of Medicine, Zhejiang University, Hangzhou, China; ^2^Key Laboratory of Kidney Disease Prevention and Control Technology, Hangzhou, China; ^3^Institute of Nephropathy, Zhejiang University, Hangzhou, China; ^4^Zhejiang Clinical Research Center of Kidney and Urinary System Disease, Hangzhou, China; ^5^Department of Nephrology, The First Affiliated Hospital of Huzhou Teachers College, The First People's Hospital of Huzhou, Huzhou, China; ^6^Zhejiang Provincial Key Laboratory for Cancer Molecular Cell Biology, Life Sciences Institute, Zhejiang University, Hangzhou, China

**Keywords:** diabetic kidney disease, metabolomics, proteinuria, biomarker discovery, progression

## Abstract

**Background:**

Diabetic kidney disease (DKD) is the primary cause of end-stage renal disease, raising a considerable burden worldwide. Recognizing novel biomarkers by metabolomics can shed light on new biochemical insight to benefit DKD diagnostics and therapeutics. We hypothesized that serum metabolites can serve as biomarkers in the progression of DKD.

**Methods:**

A cross-sectional study of 1,043 plasma metabolites by untargeted LC/MS among 89 participants identified associations between proteinuria severity and metabolites difference. Pathway analysis from differently expressed metabolites was used to determine perturbed metabolism pathways. The results were replicated in an independent, cross-sectional cohort of 83 individuals. Correlation and prediction values were used to examine the association between plasma metabolites level and proteinuria amount.

**Results:**

Diabetes, and diabetic kidney disease with different ranges of proteinuria have shown different metabolites patterns. Cysteine and methionine metabolism pathway, and Taurine and hypotaurine metabolism pathway were distinguishable in the existence of DKD in DC (diabetes controls without kidney disease), and DKD with different ranges of proteinuria. Two interesting tetrapeptides (Asn-Met-Cys-Ser and Asn-Cys-Pro-Pro) circulating levels were elevated with the DKD proteinuria progression.

**Conclusions:**

These findings underscore that serum metabolomics provide us biochemical perspectives to identify some clinically relevant physiopathologic biomarkers of DKD progression.

## Introduction

Diabetic kidney disease (DKD), accounts for 30–50% of chronic kidney disease at present, and the prevalence of which continues to increase without any sign of mitigation (1). Aside from raising the rates of end-stage renal disease (ESRD), DKD increases the risk of cardiovascular events and death (2). The natural progression of DKD is typically initiated by microalbuminuria, moves through massive proteinuria, and ends in ESRD (3, 4). Despite there having been a large amount of progress in understanding the pathogenesis of DKD, it remains challenging to distinguish type 2 diabetes (T2DM) patients who are susceptible to progressive DKD (5). Specific biomarkers are particularly urgently needed to discover individuals at risk for early identification to delay progression.

Given DKD was initiated by hyperglycemia-induced metabolic variation (6), high-throughput profiles (metabolomics) of individuals' metabolic conditions may provide biochemical perceptions related to DKD to aid in serving as possible biomarkers (7, 8). Prior studies have identified the perturbed pathways and metabolites, these studies have manifested fasting increased five branched-chain and aromatic amino acids which may help predict future type 2 diabetes development (9), lower histidine (10), and higher phenyl sulfate (11) levels were related to microalbuminuria and described remodeling of phospholipid and lipoprotein metabolism (12) and sphingolipid metabolism (13) in DKD. However, several limitations of these studies should not be ignored: (i) limited to a small set of metabolites (<200), mostly through targeted metabolomics (9, 12); (ii) lack of validation in another independent cohort (10); (iii) confined to animal models (11).

To address these problems, here we use untargeted metabolic profiling in conjunction with targeted metabolomics from healthy control, diabetes control, and diabetic kidney disease patients in a discovery set, further replicated in an independent validation set. We identified perturbed cysteine and methionine metabolism, and hypotaurine metabolism pathways in DKD patients with moderate and heavy proteinuria. Two short peptide elevations in circulation correlated with the proteinuria severity, which may serve as novel predictors of progressive diabetes.

## Materials and Methods

### Study Population for Screening

All procedures performed in this study involving human participants followed the Declaration of Helsinki. The study was approved by the Ethical Committee of the First Affiliated Hospital of Zhejiang University School of Medicine. All subjects (patients and healthy controls) provided written informed consent for blood collection.

**Figure 1:**
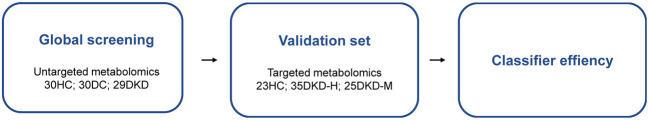
Experiment outline of this research.

The patients' study groups consisted exclusively of patients with type 2 diabetes (T2D). T2D was defined by disease onset after age 30, treated by diet and insulin or oral hypoglycaemic medication. The DKD group (n = 29) consisted of the patients with T2D and microalbuminuria (albumin 30–300 mg/day) or biopsy-proven DKD. The DKD group was further divided into two groups according to 24h-hour urine protein, or calculated as urine protein/creatinine ratio: one group consisted of 17 patients with heavy proteinuria (≥3.5 g/g), and the other group consisted of 12 patients with moderate proteinuria (<3.5 g/g). Diabetes controls without kidney disease (DC, *n* = 30) group including patients with T2D, no microalbuminuria (albumin <30 mg/day) and normal renal function (14). The healthy control (HC, *n* = 30) group consisted of people with no history of disease or current medication. Estimated glomerular filtration rate (eGFR) was evaluated using Chronic Kidney Disease Epidemiology Collaboration (CDK-EPI) equation, eGFR = 141 × min (Scr /κ, 1)^α^× max (Scr /κ, 1)^−1.209^ × 0.993^Age^ × 1.018 (if female), where: sCr is serum creatinine in mg/dL, κ is 0.7 for women and 0.9 for men, α is −0.329 for women and −0.411 for men. The clinical baseline characteristics of subjects for screening included in this study is shown in Table 1. The experimental outline as shown in Figure 1.

**Table 1:** Laboratory and clinical characteristics of individuals included in the SCREENING cohort.

	**Health control** **(*n* = 30)**	**Diabetic control** **(*n* = 30)**	**Diabetic kidney disease (DKD)**	
			**DKD with heavy proteinuria** **(*n* = 17)**	**DKD with moderate proteinuria** **(*n* = 12)**
Age (years)	50.07 ± 2.35	49.80 ± 3.33	50.18 ± 2.62	52.58 ± 3.65
Sex (male/female)	12/18	18/12	9/8	11/1
History (years)		6.40 ± 1.03	7.77 ± 1.13	7.04 ± 1.62
BMI		22.68 ± 0.73	23.15 ± 0.83	24.23 ± 1.04
SBP (mmHg)		122.90 ± 2.33	144.70 ± 5.53*	137.30 ± 6.10
DBP (mmHg)		77.17 ± 1.59	86.00 ± 2.73	86.25 ± 3.35
FPG (mmol/L)		7.66 ± 0.54	7.49 ± 0.88	6.37 ± 0.53
TC (mmol/L)		3.90 ± 0.16	5.71 ± 0.55*	4.56 ± 0.42
TG (mmol/L)		1.47 ± 0.17	1.85 ± 0.21	1.77 ± 0.29
eGFR (ml/min/1.73 m^2^)		103.70 ± 3.72	63.66 ± 7.58*	79.50 ± 17.70*
UPCR (g/g)		1.88 ± 0.13	5.72 ± 0.65*	1.81 ± 0.33

*BMI, Body Mass Index; SBP, systolic blood pressure; DBP, diastolic blood pressure; FPG, fasting plasma glucose; TC, total cholesterol; TG, triglyceride; UPCR, urine protein/creatinine ratio.*

*eGFR was evaluated using the CDK-EPI equation.*

*The data are shown as the mean ± SEM.*

* *P <0.05 vs. the health control or diabetic control*.

### Sample Collection

Peripheral venous blood samples were collected from all of the participants after 12-h fasting. After storing at 4°C for 1 h, the blood samples were centrifuged at 1,000 × g for 10 min at 4°C. The serum samples were stored in 1.5 ml Eppendorf tubes and stored at −80°C until metabolomics analysis.

### Metabolite Extraction of Serum of Screening Cohort

The serum samples were mingled with the extraction liquid (350 μl, methanol/acetonitrile/ddH_2_O, 1/2/2, v/v/v) and an internal standard (20 μl L-2-chlorophenylalanine, 1 mg/ml stock in ddH_2_O) (15). After dryness and resuspension, the supernatant was prepared for analysis by liquid chromatography (LC) mass spectrometry (MS) in Q Exactive Orbitrap (Thermo Fisher Scientific, USA).

### Metabolite Measurement of Serum by Untargeted LC/MS

Mass spectrometry (MS) was performed in both positive and negative ion modes, and the instrument parameters were as follows: the capillary voltage was 3 kV in positive mode and 2.6 kV in negative mode with the cone voltage was set at 40 V. The source and desolvation temperature were 110 and 500°C, respectively. MS analysis that simultaneously acquires both precursor and fragment mass spectra was performed on the mass spectrometer with a low collision energy of 10 eV and a ramp of 20–55 eV for high collision energy. The MS acquisition rate was set to 0.3 s with a 0.01 s interscan delay. MS data were collected in full scan mode ranging from 50 to 1,000. Nitrogen was used as both the desolvation gas (800 L/h) and the cone gas (50 L/h). The time of flight analyzer was used in Resolution mode. Leucine-enkephalin was used as the reference compound (m/z 556.2771 in ESI+ mode and m/z 554.2615 in ESI-mode) at a concentration of 200 ng/ml with a flow rate of 5 μl/min (16). Metabolites were identified and gauged based on the built-in database using Tracefinder v3.1 (Thermo Fisher Scientific, USA) based on retention times and mass-to-charge ratio.

### Study Population for Validation

The diabetic kidney disease (DKD) group (*n* = 60) included the patients with a history of T2D and presence of microalbuminuria (albumin 30–300 mg/day) or biopsy-proven DKD. The DKD group was further divided into two groups according to the Urine protein/creatinine ratio: one group consisted of 35 patients with heavy proteinuria (>3.5 g/g, *n* = 35), and the other group consisted of 25 patients with moderate proteinuria (<3.5 g/g, *n* = 25). The healthy control (HC, *n* = 23) group included the people with no history of disease or current medication.

### Metabolite Extraction of Serum of the Validation Cohort

Serum samples were prepared for LC-MS/MS analysis as described before (16). Briefly, 40 μl of each serum sample was mixed with 80 μl mass spectrometry (MS) grade methanol and incubated at 4°C. After several rounds of centrifugation at 4°C, supernatants were dried using a speed vacuum. Dried samples were re-suspended in 100 μl 60% acetonitrile/40% water. After centrifugation, 80 uL was collected and 50 uL was injected for analysis.

### Quantification of Small Peptides by Targeted LC/MS

Small peptides [Asn-Met-Cys-Ser (NS), Asn-Cys-Pro-Pro (NP)] were quantitated by LC-MS with a triple quadrupole mass spectrometer (5500 QTRAP, ABSCIEX). Peptide standards synthesized and purchased from GL Biochem were used to develop and optimize multiple reaction monitoring (MRM) transitions. Peptide standards were separated chromatographically on a C18-based column with polar embedded groups (Hypersil GOLD 100 ×2.1 mm, 3 μm, Thermo scientific) using an ABSCIEX enhanced high-performance hybrid triple quadrupole mass spectrometer with an autosampler. The flow rate was 0.4 ml/min using the following flow program: Buffer A: 99.9% H_2_O/0.1% formic acid, Buffer B: 99.9% acetonitrile/0.1% formic acid. T = 0 min, 0% B; T = 4 min, 0% B; T = 8 min, 100% B; T = 11 min, 100% B; T = 12 min, 0% B; T = 15 min, 0% B. Declustering potential, collision energy, and retention time used to detect a corresponding peptide as listed in Supplementary Table S1. Standard curves were determined, and these measured peptides exhibited good linearity between 0 and 100 μM. For each peptide, we analyzed 2–4 ion transitions. Retention time and linearity for each peptide was determined using high purity synthetic peptide standards. For all the transitions, R squares are close to 100% in curve fitting with linear regression.

### Statistical Analysis

Statistical analysis for the baseline characteristics of participants was performed using GraphPad Prism 9.0.0 software (GraphPad Inc., San Diego, CA, USA). All data are presented as the mean± SEM. Chemometrics analysis such as PCA and OPLS-DA analysis, univariate volcano plot analysis, cluster heatmap analysis was carried out by MetabAnalyst 5.0 (http://www.metaboanalyst.ca). Benjamini-Hochberg's step-down approach was used for false discovery rate correction during multiple comparisons. Normalization was carried out by using median, log transformation, and mean centering to standardize the data and make the features more comparable.

Kolmogorov-Smirnov test was used to assess for normal distribution of the data. Normally distributed data with homogeneity of variances was analyzed by using a parametric test (2 groups, unpaired student's *t* test; > 2 groups, one-way ANOVA with Holm-Šídák's multiple comparisons test). Non-normally distributed data or data with a heterogeneity of variances was analyzed by using a non-parametric test (2 groups, Mann-Whitney test. Compare ranks; > 2 groups, Kruskal-Wallis test with Dunn's multiple comparisons test). Spearman's tests were applied to determine the correlation of multiple variables with Metabolites peak area in DKD patients. A receiver operating characteristic (ROC) curve was used to evaluate the prediction value. All *P* values were two-tailed, and *P* <0.05 was considered statistically significant.

## Results

### Baseline Demographic and Clinical Characteristics of the Diabetic Patients and Healthy Controls

The baseline demographics and clinical data of the patients and controls are shown in Table 1. Urine protein/creatine ratio (UPCR) was used to calculate the amount of 24 h-urine protein and divide the diabetic kidney disease group into two groups (17), patients with heavy proteinuria (≥3.5 g/g) with an average UPCR of 5.72 g/g, and those with moderate proteinuria (<3.5 g/g) with an average UPCR of 1.81 g/g. No significant differences in age, gender ratio, BMI, DBP, FPG, or TG were observed among the three or four groups. The patients in the diabetic kidney disease (DKD) with heavy and moderate proteinuria groups presented with a mean eGFR level of 63.66 ± 7.58 ml/min/1.73 m^2^ and 79.50 ± 17.70 ml/min/1.73 m^2^, respectively (*P* <0.05 compared with the diabetic controls).

### Serum Metabolic Profiles Can Differentiate Between DKD From DC

**Figure 2:**
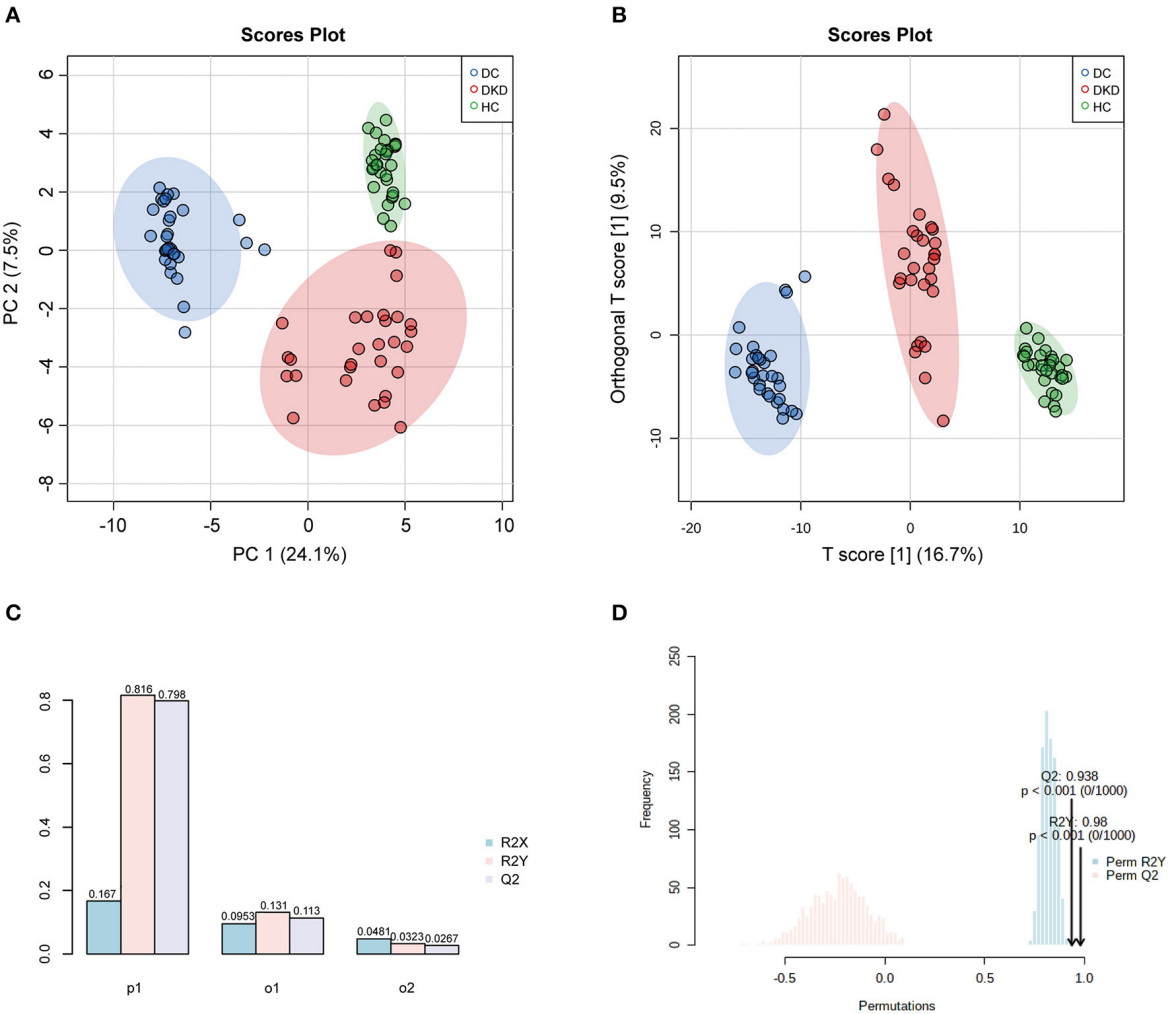
Overall similarity and differences between samples by PCA and OPLS-DA analysis. **(A)** PCA score plots of healthy controls (HC), diabetic controls (DC), diabetic kidney disease patients [DKD, i.e., Diabetic nephropathy (DN)]. **(B,C)** OPLS-DA score and model overview plots of HC, DC, and DKD. **(D)** 1,000-times permutation test of the model showed its high strong reliability.

A clear separation between healthy controls (HC), diabetic controls (DC), and diabetic kidney disease patients (DKD) was displayed in the unsupervised Principal Component Analysis (PCA), with PC1 at 24.1% and PC2 at 7.5% (Figure 2A). The significant distinction in metabolic profiles was further supported by Orthogonal PLS-DA (OPLS-DA) model, with the R2Ycum = 81.6%, Qcum2 = 79.8% (Figures 2B,C). These results indicated diabetic kidney disease development alters the serum metabolite landscape of diabetes, so the serum metabolic signatures can be appropriate to distinguish between DKD from DC. Furthermore, the permutation test showed the high stability of the model (Figure 2D).

### Differences of the Serum Metabolites Within Each Group

On the whole, 1,043 metabolites were relatively quantified using LC/MS after strict quality control, 368 metabolites had >20% missing observations were excluded from the analysis. An overview metabolite profiles heatmap between three groups, ie. healthy controls (HC), diabetic controls (DC), diabetic kidney disease patients (DKD) is shown in Supplementary Figure S1. As described in Materials and Methods, through a moderated t-test with *P*-value corrected applying the Benjamini-Hochberg procedure, differentially expressed metabolites (DEMs) of these groups were defined as metabolites with a significance level of <0.05 (FDR) and absolute fold-change >1.5 or < 0.67, we obtained the differentially expressed metabolites (DEMs) of these groups.

**Figure 3:**
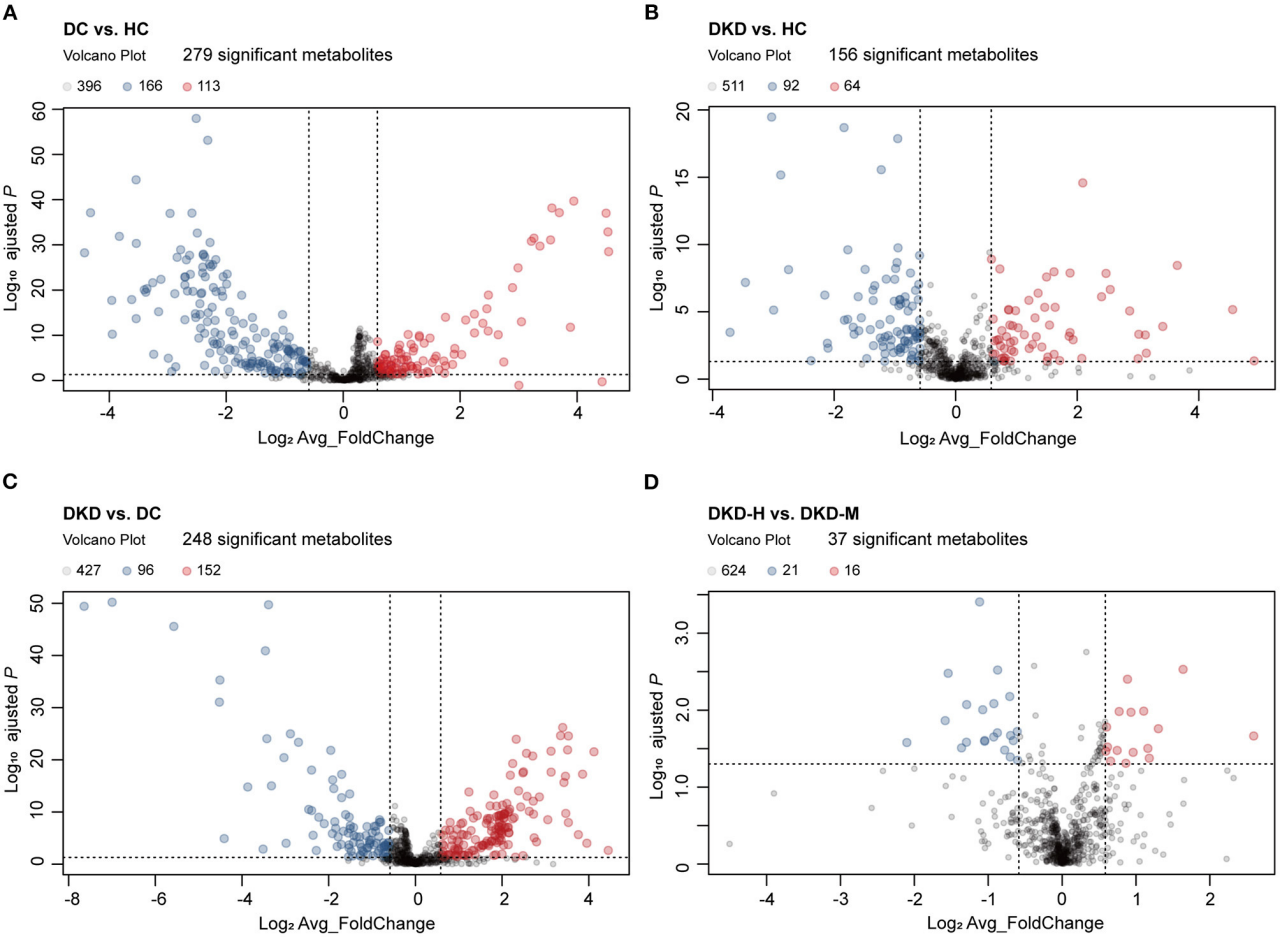
Visualization of serum metabolites difference between healthy controls and diabetic patients. **(A)** Volcano plot comparing serum metabolites in diabetic controls (DC) (*n* = 30) and healthy controls (HC) (*n* = 30). The vertical dashed lines indicate the threshold for the 1.5-fold abundance difference. The horizontal dashed line indicates the *P* = 0.05 threshold. X-axis, log2[average_FoldChange]. Y-axis, –log10[adjusted-*P* value]. *P*-value computed using a two-sided unpaired t-test without adjustment for multiple comparisons. **(B)** Volcano plot comparing serum metabolites in diabetic kidney disease patients (DKD) (*n* = 29) and healthy controls (HC) (*n* = 30). Refer to **(A)** for the description of the figure. **(C)** Volcano plot comparing serum metabolites in diabetic kidney disease patients (DKD) (*n* = 29) and diabetic controls (DC) (*n* = 30). Refer to (A) for the description of the figure. **(D)** Volcano plot comparing serum metabolites in diabetic kidney disease with heavy proteinuria (DKD-heavy) (*n* = 17) and diabetic kidney disease with moderate proteinuria (DKD-moderate) (*n* = 12). Refer to **(A)** for the description of the figure.

Of these, 113 metabolites were significantly upregulated and 166 metabolites were significantly downregulated between DC and HC groups (Figure 3A). Similarly, there were 64, 152, and 16 metabolites that had been noticed to be upregulated significantly and 92, 96, 21 metabolites downregulated significantly in DKD vs. HC group, DKD vs. DC group, and DKD-heavy vs. DKD-moderate group, respectively (Figures 3B–D). The detailed list of differential metabolites within each group including name, fold change, *P*-value, HMDB number, and KEGG ID are provided in Supplementary Table S2.

### Disturbed Cysteine and Methionine Metabolism and Hypotaurine Metabolism Pathway

**Figure 4:**
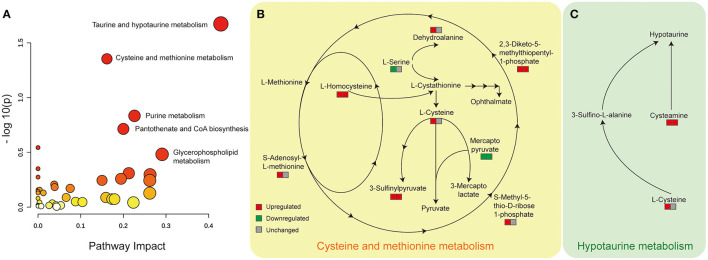
Disturbed cysteine and methionine metabolism and hypotaurine metabolism pathway with significance identified from Metabanalyst in the comparison of DKD vs. DC, DKD-H vs. DKD-M. **(A)** Disturbed metabolic pathways were identified from the changed metabolites from the comparison of DKD vs. DC and DKD-H vs. DKD-M using serum samples. All matched pathways according to the *p* values from the pathway enrichment analysis and pathway impact values from the pathway topology analysis. The color of each node (varying from yellow to red) means the metabolites are in the data with different levels of significance, the size of each node represents the pathway impact values. **(B)** Altered serum metabolites in the cysteine and methionine metabolism pathway. The left square refers to the comparison of DKD vs. DC, the right square refers to the comparison of DKD-H vs. DKD-M. Red means upregulated more than 1.1 fold, Green means downregulated less than 0.91 fold, and gray means unchanged whose range between 0.91 and 1.1 in each comparison. **(C)** Altered serum metabolites in the hypotaurine metabolism pathway. Refer to **(B)** for the description of the figure.

Aiming to understand the perturbed metabolism pathway completely, we firstly used loose criterion (>1.1 fold or <0.91 fold) to screen the differential metabolites in the comparison of diabetic kidney disease (DKD) vs. diabetic control (DC) and diabetic kidney disease with heavy proteinuria (DKD-H) vs. diabetic kidney disease with moderate proteinuria (DKD-M). That is to say, to identify disturbed pathways that perturbed in the onset of DKD, and altered continuously in the progression of DKD. Among the identified enriched pathways from the differential metabolites, the hypotaurine metabolism pathway, cysteine, and methionine metabolism pathway were found with the significance of *p* <0.05 (Figure 4A). Serum L-homocysteine and 3-sulfinylpyruvate, and 2,3-Diketo-5-methythiopentyl-1-phosphate seem to increase when DKD exists in the set of diabetes and increase with the proteinuria progression of DKD (Figure 4B). Although dehydroalanine and L-cysteine, s-adenosyl-L-methionine, and s-methyl-5-thio-D-ribose 1-phosphate were found to be raised in DKD compared to DC, almost no difference was found in the two different ranges of proteinuria groups. Mercaptopyruvate was reduced in the set of diabetic kidney disease and further decreased in the heavy proteinuria group. The Hypotaurine metabolism pathway was the other perturbed pathway identified with significance in our comparison (Figure 4C). Taurine, also called amino sulfonic acid, whose antioxidant property and protective role in many kidney diseases were widely reviewed (18, 19), while hypotaurine is an intermediate of taurine synthesis, it oxidated to taurine through hypotaurine dehydrogenase. No differences were observed between DKD vs. DKD-H vs. DKD-M (data not shown). However, due to the limitation of metabolites detected by our mass spectrometry, we lack information on serum hypotaurine alternation.

### Two Short Peptides Highly Correlated With the Progression of DKD

**Figure 5:**
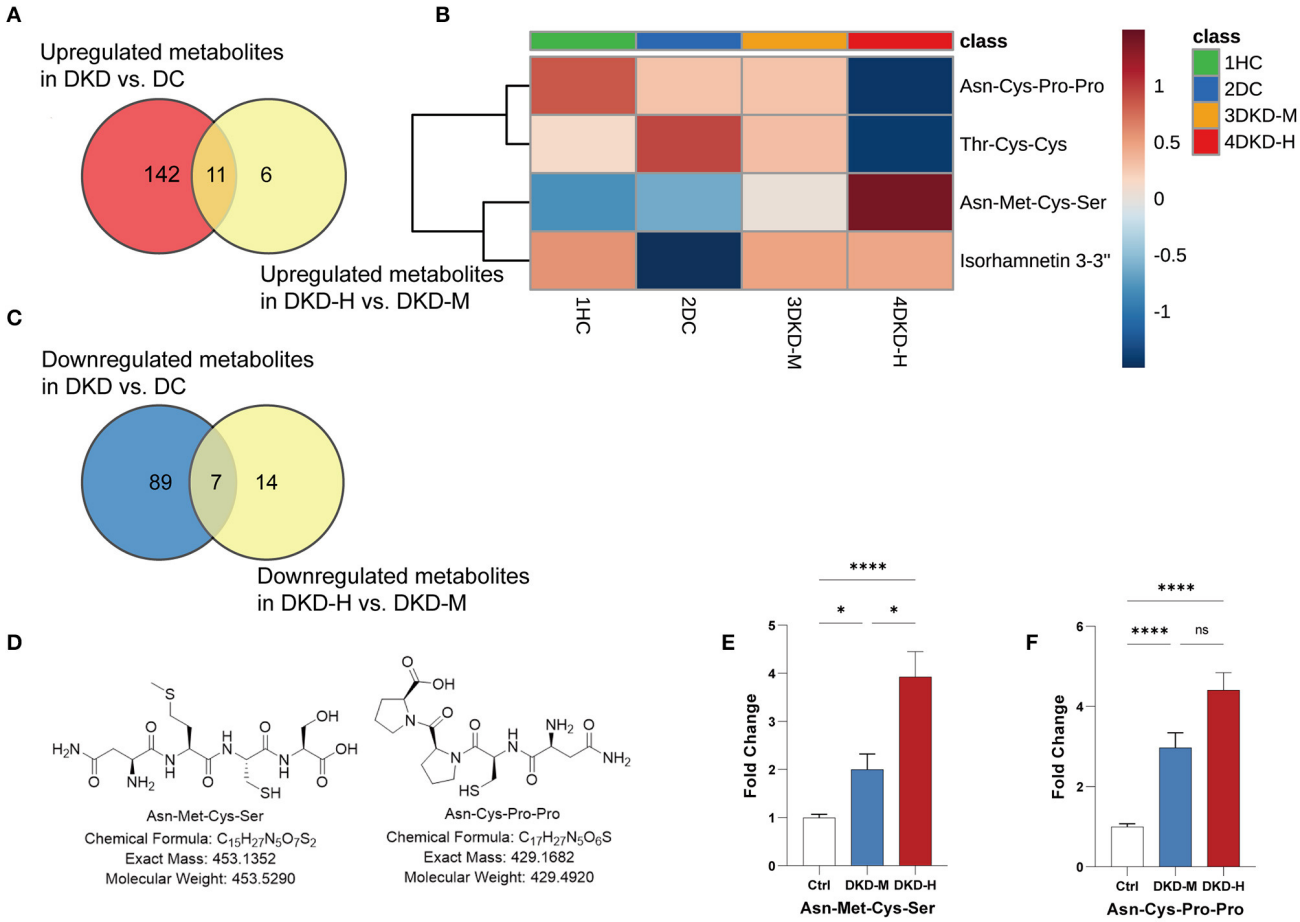
Asn-Met-Cys-Ser and Asn-Cys-Pro-Pro peak count changed with the progression of diabetic kidney disease. **(A)** Venn diagrams showing the number of upregulated (fold change ≥ 1.5) metabolites in DKD vs. DC and DKD-heavy vs. DKD-moderate (*p* < 0.05). **(B)** Venn diagrams showing the number of downregulated (fold change ≤ 0.67) metabolites in DKD vs. DC and DKD-heavy vs. DKD-moderate (*p* < 0.05). **(C)** Heatmap of 4 metabolites, Asn-Met-Cys-Ser, Asn-Cys-Pro-Pro, Thr-Cys-Cys and Isorhamnetin 3-(3″, 6″-di-p-coumarylglucoside) changed significantly (fold change ≥ 2 or ≤ 2) in the same direction in the comparison of DKD vs. DC and DKD-heavy vs. DKD-moderate. **(D)** Chemical structural formula, exact mass, and molecular weight of Asn-Met-Cys-Ser and Asn-Cys-Pro-Pro. **(E,F)** Confirmation of peak counts of Asn-Met-Cys-Ser and Asn-Cys-Pro-Pro in the validation group. Healthy control (HC) (*n* = 23), diabetic kidney disease with moderate proteinuria (DKD-M) (*n* = 25), and diabetic kidney disease with heavy proteinuria (DKD-H) (*n* = 35). The data are shown as the mean ± SEM. *P < 0.05 vs. the corresponding control group.

During the process of the above pairwise comparison, to find metabolites associated with the progression of DKD, we focused on the two comparisons, DKD vs. DC and DKD-heavy vs. DKD-moderate, in purpose to detect who was susceptible to kidney disease in diabetes condition and who were vulnerable to progressive proteinuria under DKD. Eleven collective metabolites were significantly upregulated in DKD compared to DC and in DKD-H compared to DKD-M, and seven common metabolites that downregulated in DKD compared to DC and in DKD-H compared to DKD-M were found (fold change > 1.5) (Figures 5A,B).

To simplify the metabolite biomarker panel for possible clinical application, we concentrated on the metabolites that: (1) have commercially available reference compounds and (2) have a higher difference in the two comparisons (fold change>2 or fold change <0.5). Among the 4 metabolites (Table 2), Asn-Met-Cys-Ser, Asn-Cys-Pro-Pro, Thr-Cys-Cys, and Isorhamnetin 3-(3″,6″-di-p-coumarylglucoside), the first two peptides were finally chosen as possible targets to predict DKD progression (Figures 5C,D).

**Table 2:** Four metabolites in the same direction after successive comparison.

**Name**	**log2** $\frac{DKD}{DC}$	***P*-value**	**log2** $\frac{DKD-H}{DKD-M}$	***P*-value**
Asn-Met-Cys-Ser	1.1261	1.71E−02	2.5934	2.17E−02
Asn-Cys-Pro-Pro	1.7428	3.00E−02	1.9677	4.55E−02
Thr-Cys-Cys	−1.5338	2.04E−04	−1.0485	2.54E−02
Isorhamnetin 3-(3″,6″-di-p-coumarylglucoside)	1.51	7.46E−04	1.303	1.74E−02

Twenty-three healthy controls and sixty diabetic kidney disease patients were recruited for further validation. The baseline demographics and clinical data of the patients and controls in verification group are shown in Table 3. Their blood was collected for targeted metabolomics analysis. Differences in Asn-Met-Cys-Ser and Asn-Cys-Pro-Pro levels between DKD-heavy patients and DKD-moderate patients remained statistically significant (Figures 5E,F).

**Table 3:** Characteristics of individuals included in the VERIFICATION group.

		**Diabetic kidney disease (DKD)**	
	**Health control** **(*n* = 23)**	**DKD with** **heavy proteinuria** **(*n* = 35)**	**DKD with** **moderate proteinuria** **(*n* = 25)**
Age (years)	44.57 ± 2.59	51.37 ± 1.73	52.04 ± 2.22
Sex (male/female)	10/13	26/9	20/5
History (years)		7.59 ± 0.94	6.93 ± 1.08
BMI		24.13 ± 0.55	23.61 ± 0.45
SBP (mmHg)		150.00 ± 3.61	144.00 ± 2.93
DBP (mmHg)		87.63 ± 1.79	85.96 ± 1.97
FPG (mmol/L)		8.19 ± 0.62	6.96 ± 0.62
HbA1c (%)		7.43 ± 0.34	7.40 ± 0.33
TC (mmol/L)		5.27 ± 0.26*	4.27 ± 0.20
TG (mmol/L)		2.18 ± 0.23	1.89 ± 0.22
eGFR (ml/min/1.73 m^2^)		58.49 ± 5.70	60.60 ± 5.11
UPCR (g/g)		6.97 ± 0.43*	2.16 ± 0.16

*BMI, Body Mass Index; SBP, systolic blood pressure; DBP, diastolic blood pressure; FPG, fasting plasma glucose; TC, total cholesterol; TG, triglyceride; UPCR, urine protein/creatinine ratio.*

*eGFR was evaluated using the CDK-EPI equation.*

*The data are shown as the mean ± SEM.*

* *P <0.05 vs. the control or DKD with moderate proteinuria group*.

### Association Between Serum Asn-Met-Cys-Ser and Asn-Cys-Pro-Pro Intensities and the Presence of DKD

**Figure 6:**
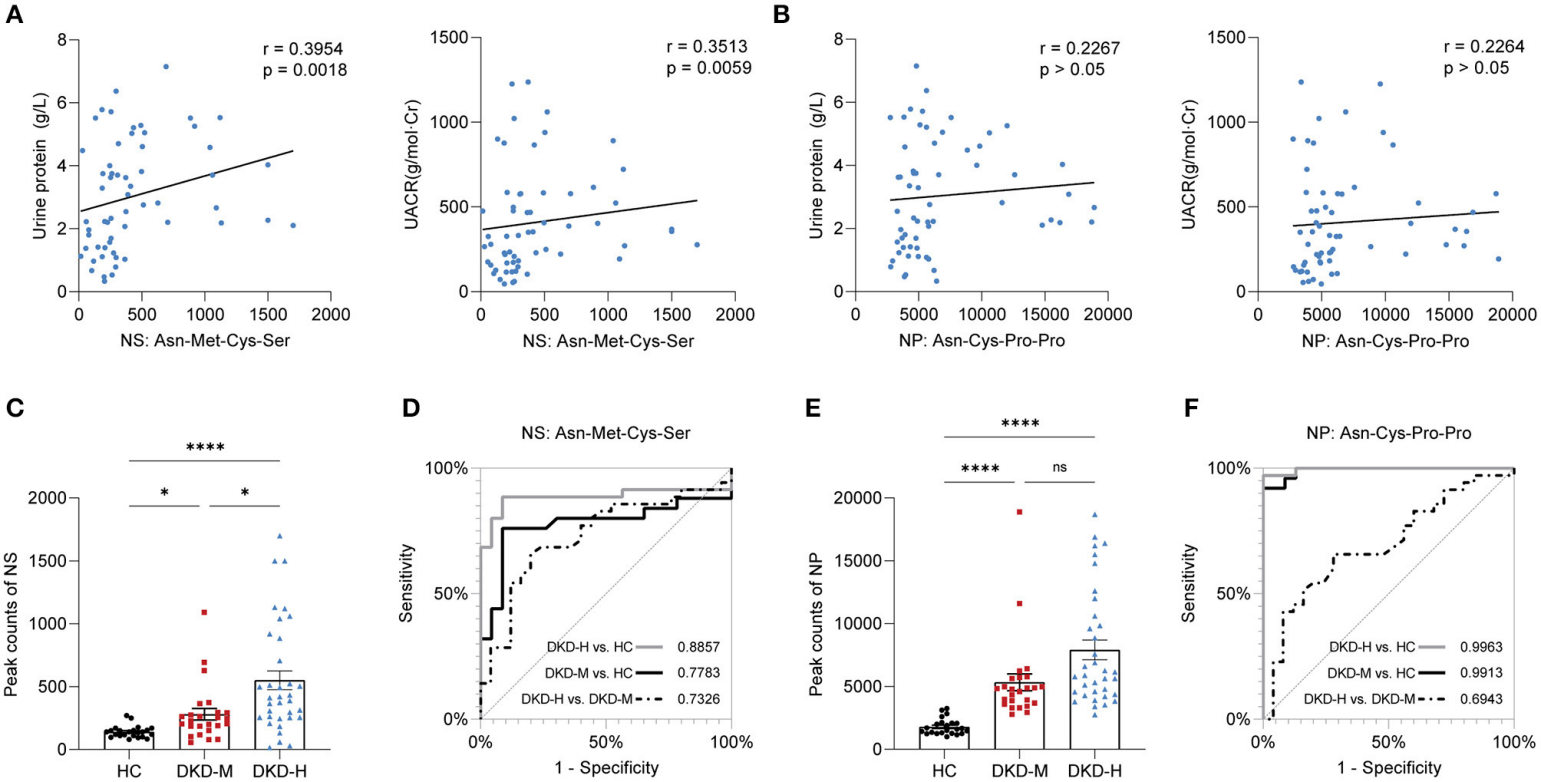
Correlation with clinical parameters and prediction value. **(A)** Correlation of urine protein (g/L) and UACR (g/mol·Cr) with Asn-Met-Cys-Ser. **(B)** Correlation of urine protein (g/L) and UACR (g/mol·Cr) with Asn-Cys-Pro-Pro. **(C)** Individual value plots of Asn-Met-Cys-Ser in the validation group. **(D)** Area under the curve (AUC) of prediction models based on Asn-Met-Cys-Ser. **(E)** Individual value plots of Asn-Cys-Pro-Pro in the validation group. **(F)** Area under the curve (AUC) of prediction models based on Asn-Cys-Pro-Pro. The data are shown as the mean ± SEM. *P < 0.05 vs. the corresponding control group.

Correlation analysis showed Asn-Met-Cys-Ser was correlated with urine protein (g/L) and UACR (g/mol·Cr) (Figure 6A, all *p* <0.01), while Asn-Cys-Pro-Pro was independent of urine protein and UACR level in DKD patients (Figure 6B). Other clinical parameters, such as age, sex, blood pressure, HbA1c, serum lipids, eGFR, sCr, BUN, and Ucr were unconnected with Asn-Met-Cys-Ser and Asn-Cys-Pro-Pro levels between DKD patients (Supplementary Figures S2, S3).

The result indicated that serum Asn-Met-Cys-Ser and Asn-Cys-Pro-Pro levels were progressively increased in the development of DKD (Figures 6C,E). To examine the performance of two metabolites in the prediction of DKD, ROC curves were developed. The results of ROC curves revealed that the best cutoff value for circulating Asn-Met-Cys-Ser to predict DKD with heavy proteinuria in DKD patients was 14.27 nM (sensitivity: 80%, specificity: 65.7%) (Figure 6D). The results of ROC curves revealed that the best cutoff value for circulating Asn-Cys-Pro-Pro to predict DKD with moderate proteinuria was 1.03 M (sensitivity: 92%, specificity: 85.69%) (Figure 6F).

## Discussion

As the leading cause of chronic kidney disease (CKD), diabetic kidney disease (DKD) brings a substantial burden worldwide (1). It is urgent to identify which at-risk individuals are extremely likely to develop progressive diabetic kidney disease. Over the past decades, plenty of studies have used mass spectrometry for biomarker discovery in diabetic kidney disease (8, 10–13), but these studies have been largely limited to a narrow range of metabolites, unable to depict the full view of metabolite profiles. Proteinuria, a marker of diabetic kidney disease, as well as an independent risk factor of renal disease progression, can cause tubulointerstitial injury to reduce eGFR in DKD (20). Furthermore, the relationship between proteinuria severity and metabolite profiling in diabetes patients has not been investigated yet. Hence, an advantage of this study is the use of untargeted metabolomics involved 1,043 various metabolites combined with targeted metabolomics in two independent cohorts, one for screening and one for validation. Utilizing mass spectrometry-based metabolomics, we identified two disturbed pathways and picked out two oligopeptides whose fasting blood concentrations highly correlated with the forthcoming development of progressive DKD in diabetic individuals.

During the pathway analysis of differentially expressed metabolites (DEMs), cysteine and methionine metabolism pathway and hypotaurine metabolism pathway have drawn our attention. Although circulating cysteine itself elevated 1.5-fold compared DKD to DC, and does not seemto show much alteration between the two different proteinuria groups of DKD, it must be taken into account that being “unchanged” may be an illusion caused by alteration in the same direction in the synthesis and breakdown. Homocysteine was widely researched in the past decades, whether it is an independent risk factor in DKD is controversial (21). Homocysteine is converted from methionine transmethylation, as S-adenosyl-L-methionine (SAM) and S-adenosylhomocysteine as the intermediate product. The homocysteine metabolism has twomethods, being remethylated with the methyl of 5-methyl TNF *via* the methionine cycle, or turned into cysteine *via* the transsulfuration pathway (22). The elevated circulating SAM, homocysteine, cysteine, and reduced serine were in accordance with the previous investigation about renal insufficiency (23), these perturbed metabolism may be caused by the disruption of methionine transmethylation and cysteine metabolism reprogramming or as the result of kidney function decline. A series of stable isotope-labeled metabolomics techniques certificated the methionine cycle flux decreased 30% compared to healthy controls with the methionine transmethylation and remethylation pathway were reduced in ESRD patients (24, 25).

Disturbed taurine and hypotaurine metabolism pathway in STZ-induced diabetic mice was discovered by Nuclear magnetic resonance (NMR) spectroscopy (26). Taurine, as one of the few amino acids which does not build protein, was found to play a role in adjusting renal blood flow, clearing ROS in the glomerulus, transporting Na+ in the kidney proximal tubule, and regulating osmotic pressure in the renal medulla (18). However, whether a taurine supplement will ameliorate T2DM development was full of debate in animal models and diabetic patients over the past decades (19, 27).

**Figure 7:**
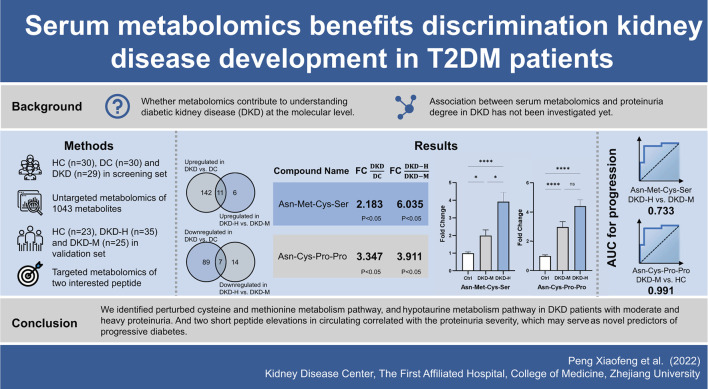
Schematic illustration of the present study.

On the other hand, in order to find a novel latent marker, we focused on the DEMs strikingly altered (FoldChange ≥2 and *p*-value <0.05) in both comparisons of DKD vs. DC and DKD-H vs. DKD-M. Finally, two tetrapeptides, Asn-Met-Cys-Ser and Asn-Cys-Pro-Pro were chosen in the next validation. In another population made up of 23 healthy controls, 35 DKD with heavy proteinuria patients and 25 DKD with moderate proteinuria patients, serum Asn-Met-Cys-Ser levels were correlated with the severity of proteinuria, while Asn-Cys-Pro-Pro seems similar in the two sets of proteinuria group. Dietary protein does not need to be hydrolyzed completely into amino acids before they are absorbed in the gastrointestinal tract. Food digestion produces a huge number of short peptides (oligopeptides), every possible di-peptides and tri-peptides digested by peptidases can enter into intestinal endothelium *via* the PepT1 transporter and have access to the hepatic portal system (28). While oligopeptides contain four or more amino acids entering into the intestine through transcytosis, or paracellular pathway *via* intracellular junction (29, 30). Asn-Cys-Pro-Pro with the proline-proline at the C' terminal, which leads to digestion resistance by proteases and peptidases (31). These two oligopeptides escape hydrolysis, and were easy to clear by renal excretion theoretically, their circulating levels increased with proteinuria amount indicating they may serve as a bioactive peptide and exert effects in the DKD progression. Correlation analysis and ROC curve analysis showed Asn-Met-Cys-Ser and Asn-Cys-Pro-Pro have the potential to distinguish the severity of DKD patients. A graphic abstract is shown in Figure 7.

The results of the present study manifest that circulating metabolite can be an effective tool to provide us brand-new perspective in understanding biochemical alteration in patients with DKD. Disturbed cysteine and methionine metabolism and hypotaurine metabolism were companied with the DKD, and two oligopeptides, Asn-Met-Cys-Ser and Asn-Cys-Pro-Pro validated in another cohort, which may serve as a promising marker for selecting DKD patients who are at high risk to progression. These findings for disturbed metabolites and metabolism pathways can help us to pinpoint novel targets for treating individuals with early progressive DKD and delay the progression to ESRD.

To our knowledge, this was the first study to show the relation of serum metabolites profiling and proteinuria severity in DKD, which may provide a different insight to treat the disease. Nonetheless, there are some imitations. Owing to the high dynamic of metabolomics, the direction of impact was hard to ascertain (32). We show the homocysteine was increased with the severity of proteinuria, but the exact mechanism is unknown. Secondly, we have validated only two metabolites targets, and how the two oligopeptides correlated with proteinuria severity remains unstudied.

## Data Availability Statement

The datasets presented in this study can be found in online repositories. The names of the repository/repositories and accession number(s) can be found below: https://figshare.com/s/02319eeb01c363a87928.

## Ethics Statement

The studies involving human participants were reviewed and approved by Ethical Committee of the First Affiliated Hospital of Zhejiang University School of Medicine. The patients/participants provided their written informed consent to participate in this study.

## Author Contributions

XP, XS, and HJ designed this project. XP and XW performed experiments with help of YW, SF, and CW. CY, XP, and XS prepared the figures and wrote the manuscript. JC and HJ supervised work and acquired funding. All authors participated in discussions about this study and approved the final version to be published.

## Funding

This work was supported by grants from the National Key Research and Development Plan (2018YFC1314003).

## Conflict of Interest

The authors declare that the research was conducted in the absence of any commercial or financial relationships that could be construed as a potential conflict of interest.

## Publisher's Note

All claims expressed in this article are solely those of the authors and do not necessarily represent those of their affiliated organizations, or those of the publisher, the editors and the reviewers. Any product that may be evaluated in this article, or claim that may be made by its manufacturer, is not guaranteed or endorsed by the publisher.

## Supplementary Material

Supplementary Figure S1 A overview 675 metabolite heatmap between three groups, ie. healthy controls (HC), diabetic controls (DC), diabetic kidney disease patients (DKD). Each line of this graphic represents a relation of different metabolites, colored by their relative intensities (red, upregulated; blue, downregulated). Columns: serum sample groups; rows: metabolites. Each point represents the average metabolite peak counts in each group.

Supplementary Figures S2, S3 Asn-Met-Cys-Ser and Asn-Cys-Pro-Pro were independent of age, sex, SBP, DBP (mmHg), HbA1c (%), TC (mmol/L), TG (mmol/L), eGFR (ml/min/1.73 m^2^), sCr (μmol/L), BUN (mmol/L), Ucr (mmol/L).

Supplementary Table S1 Declustering potential, collision energy, and retention time of two peptides.

Supplementary Table S2 Different metabolites in each comparison.
