# Supplementary figures and images for: Serum Metabolomics Benefits Discrimination Kidney Disease Development in Type 2 Diabetes Patients
Source: Front Med (Lausanne). 2022 May 9;9:819311. doi: 10.3389/fmed.2022.819311 (PMC9126316; doi:10.3389/fmed.2022.819311)

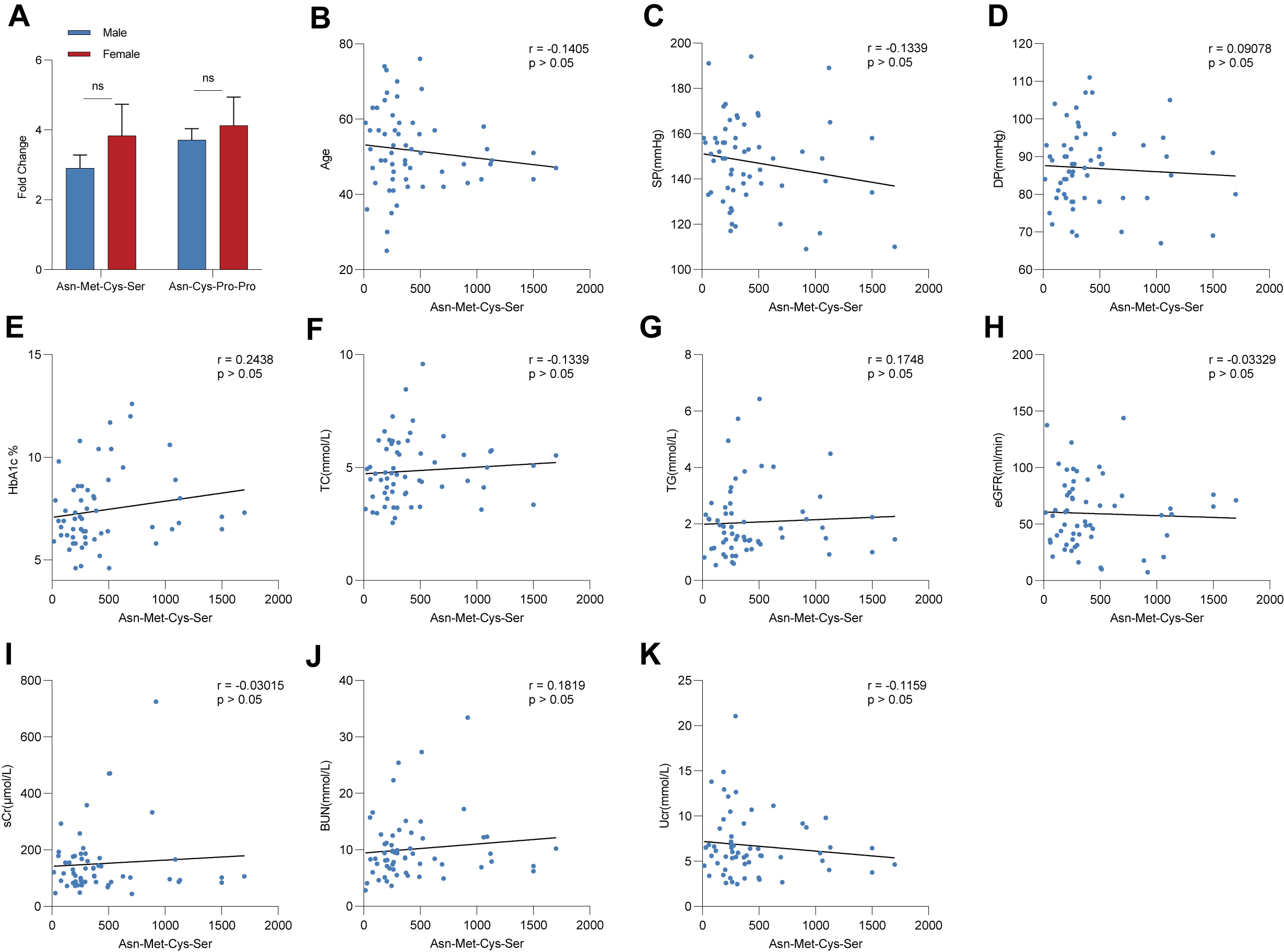

Supplement: Supplementary file 2 [file Image_2.TIF]

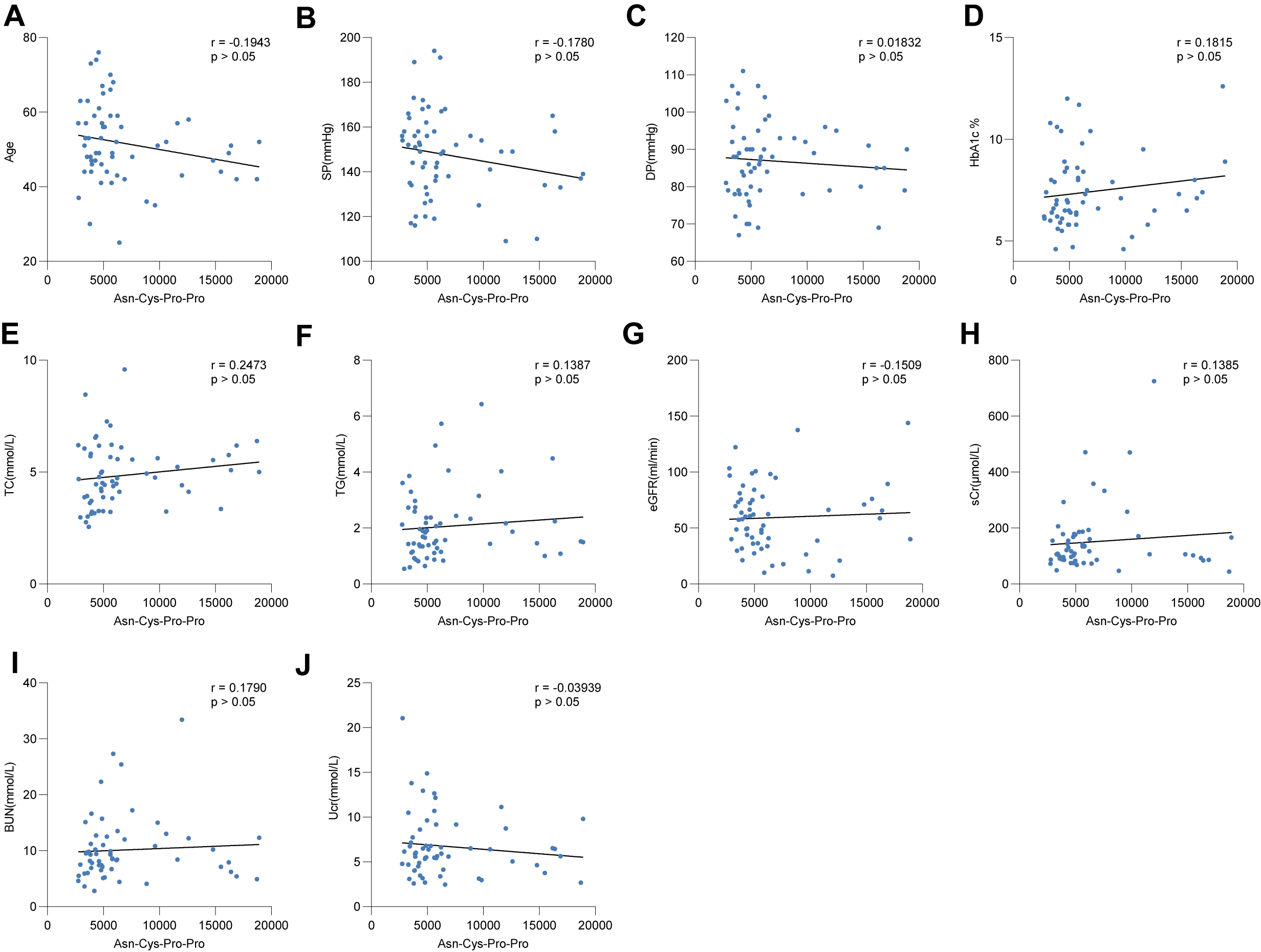

Supplement: Supplementary Figures S2, S3 — Asn-Met-Cys-Ser and Asn-Cys-Pro-Pro were independent of age, sex, SBP, DBP (mmHg), HbA1c (%), TC (mmol/L), TG (mmol/L), eGFR (ml/min/1.73 m2), sCr (μmol/L), BUN (mmol/L), Ucr (mmol/L). [file Image_3.TIF]
